# FibroAgent: An Agentic AI Tool for Liver Fibrosis Screening and Clinical Decision Support in Metabolic Dysfunction-Associated Steatotic Liver Disease (MASLD)

**DOI:** 10.7759/cureus.106372

**Published:** 2026-04-03

**Authors:** Basile Njei, Issa Ali, Yazan Al-Ajlouni, Sarpong Boateng, Guy Loic Nguefang, Prince Ameyaw, Ulrick Sidney Kanmounye, Nelvis Njei, Solomon Gyabaah

**Affiliations:** 1 Medicine, Yale School of Medicine, New Haven, USA; 2 Internal Medicine, Cleveland Clinic Florida, Weston, USA; 3 School of Medicine, New York Medical College, New York, USA; 4 Medicine, Yale New Haven Health, Bridgeport Hospital, New Haven, USA; 5 Internal Medicine, Texas Tech University Health Sciences Center, Odessa, USA; 6 Neurosurgery, Bel Campus University of Technology, Kinshasa, COD; 7 Global Neurosurgery Initiative, Harvard Medical School, Boston, USA; 8 Pharmacology, Centers for Machine Learning Intelligence (M-LINT), Elcott City, USA; 9 Internal Medicine, Komfo Anokye Teaching Hospital, Kumasi, GHA

**Keywords:** agentic ai, fibroagent, liver fibrosis, liver fibrosis screening, masld

## Abstract

Background

Metabolic dysfunction-associated steatotic liver disease (MASLD) affects approximately one-quarter of adults worldwide, yet liver fibrosis remains markedly under-recognized in primary care. Existing screening approaches using static risk calculators yield single numeric outputs without explanation, actionable recommendations, or supporting documentation, limiting their adoption and clinical impact.

Methods

We developed FibroAgent, a conversational agentic AI framework that integrates a validated machine-learning XGBoost-based prediction model, patient-specific explainability via SHapley Additive exPlanations (SHAP), risk-stratified clinical pathways, an interactive educational knowledge base, and structured documentation within a single autonomous agent. FibroAgent was designed following established agentic AI principles: proactiveness, autonomy, reactivity, and transparency, and requires only seven routinely available clinical parameters (age, glycated hemoglobin (HbA1c), alanine aminotransferase (ALT), aspartate aminotransferase (AST), platelet count, body mass index (BMI), and glomerular filtration rate (GFR)). We demonstrated its capabilities across low-risk, intermediate-risk, and high-risk patient scenarios, batch screening of a six-patient cohort, and error-handling validation.

Results

FibroAgent correctly stratified patients with advanced liver fibrosis into rule-out (probability <=0.20), indeterminate (0.20-0.69), and rule-in (>=0.70) categories with corresponding risk-appropriate clinical recommendations. SHAP-based explanations identified platelet count, AST, and age as the top-three risk contributors in high-risk patients, providing a clinically interpretable rationale for each prediction. The agent demonstrated robust error handling, maintaining analytical continuity despite simulated tool failures.

Conclusions

FibroAgent represents a novel and promising approach to transitioning from static fibrosis calculators to interactive, explainable, and autonomous clinical decision support. By unifying prediction, explanation, recommendation, education, and documentation within a single conversational agent, FibroAgent offers a scalable, imaging-independent triage solution for primary care and resource-limited settings.

## Introduction

Metabolic dysfunction-associated steatotic liver disease (MASLD) has emerged as one of the most prevalent chronic liver diseases worldwide, affecting an estimated one-quarter to one-third of adults, with substantially higher prevalence among individuals with type 2 diabetes and other cardiometabolic risk factors [1,2]. Despite its high frequency and well-established associations with advanced fibrosis, cirrhosis, hepatocellular carcinoma, cardiovascular disease, and premature mortality, MASLD remains markedly underdiagnosed, particularly in primary care and community-based settings [1,3]. A major driver of this gap is the silent progression of liver fibrosis, coupled with inconsistent screening practices and limited access to scalable, non-invasive diagnostic tools outside of speciality hepatology care.

Multiple international policy and expert consensus documents have emphasized that effective liver disease prevention depends on shifting fibrosis detection upstream, from tertiary hepatology clinics to primary care [1,2,4]. The Lancet Commission into Liver Disease in the UK highlighted that most patients with advanced fibrosis are identified too late for meaningful intervention and recommended a two-tier screening strategy using simple blood-based risk scores, such as Fibrosis-4 (FIB-4) or the Nonalcoholic Fatty Liver Disease (NAFLD) Fibrosis Score, followed by transient elastography for those at higher risk [1]. However, the Commission also acknowledged that mobilizing primary care to implement such pathways remains challenging due to structural, logistical, and resource constraints [1].

Fewer than 10% of affected patients are referred to specialist services even in high-income health systems, reflecting low confidence among primary care providers in identifying and risk-stratifying chronic liver disease [2,5]. Although vibration-controlled transient elastography (FibroScan) is a validated and effective second-tier test for detecting advanced fibrosis, its availability remains uneven, with access dependent on local expertise, equipment, and referral infrastructure [4]. Consequently, reliance on elastography alone has not resolved disparities in early fibrosis detection.

These limitations are further amplified in low-resource and low- and middle-income settings [3]. Policy-focused reviews from sub-Saharan Africa and other under-resourced regions highlight that while FibroScan is portable and can be operated by non-physician healthcare workers, cost and limited distribution have constrained its integration into routine primary care [3]. As a result, large segments of the global population remain without access to timely fibrosis assessment, perpetuating inequities in liver-related outcomes [3,6].

Population-based screening studies underscore the magnitude of missed disease. Approximately 5% of the general population and up to 18-27% of individuals with metabolic risk factors are estimated to harbor undiagnosed liver fibrosis [6]. While blood-based non-invasive tools, such as FIB-4, are inexpensive and universally available, their high indeterminate rates, limited specificity, and variable performance across diverse populations restrict their utility as standalone screening instruments and often fail to provide clear clinical direction for frontline clinicians [2,5].

Together, these data highlight a critical unmet need for scalable, low-cost decision-support tools that can bridge the gap between population-level screening and specialist referral, particularly in primary care and resource-limited environments [4,6]. Artificial intelligence-based models offer the potential to overcome key limitations of traditional fibrosis scores by capturing nonlinear relationships among routinely collected clinical variables while remaining independent of imaging infrastructure [2,4,7]. However, for successful real-world adoption, such tools must be transparent, interpretable, and capable of supporting, not replacing, clinical decision-making [4].

Agentic clinical decision support represents a fundamentally different paradigm from standalone prediction models. Rather than producing a single numerical output, an agentic system can interact with users, contextualize predictions, provide explanations, educate clinicians and patients, guide workflows, and generate documentation within a unified interface [5]. In this proof-of-concept paper, we present FibroAgent, a deployable conversational clinical decision support (CDS) framework that integrates a validated machine-learning XGBoost model, patient-specific explainability via SHapley Additive exPlanations (SHAP; https://shap.readthedocs.io/en/latest/index.html), risk-based clinical pathways, an educational knowledge base, and structured report generation into a single interactive agent. The primary objective of this study is to describe the development and provide a proof-of-concept demonstration of FibroAgent as an agentic clinical decision support framework for risk stratification of advanced liver fibrosis in MASLD using routinely available clinical parameters. Secondary objectives are to demonstrate the integration of explainable AI through SHAP-based feature attribution, illustrate workflow integration within an agentic clinical decision support framework, and demonstrate feasibility across representative clinical scenarios and batch screening applications. We describe its conceptual framework through the lens of agentic AI design principles, demonstrate its capabilities across representative clinical scenarios, and discuss the implications for scalable fibrosis detection in MASLD.

## Materials and methods

Agentic AI design framework

**Figure 1:**
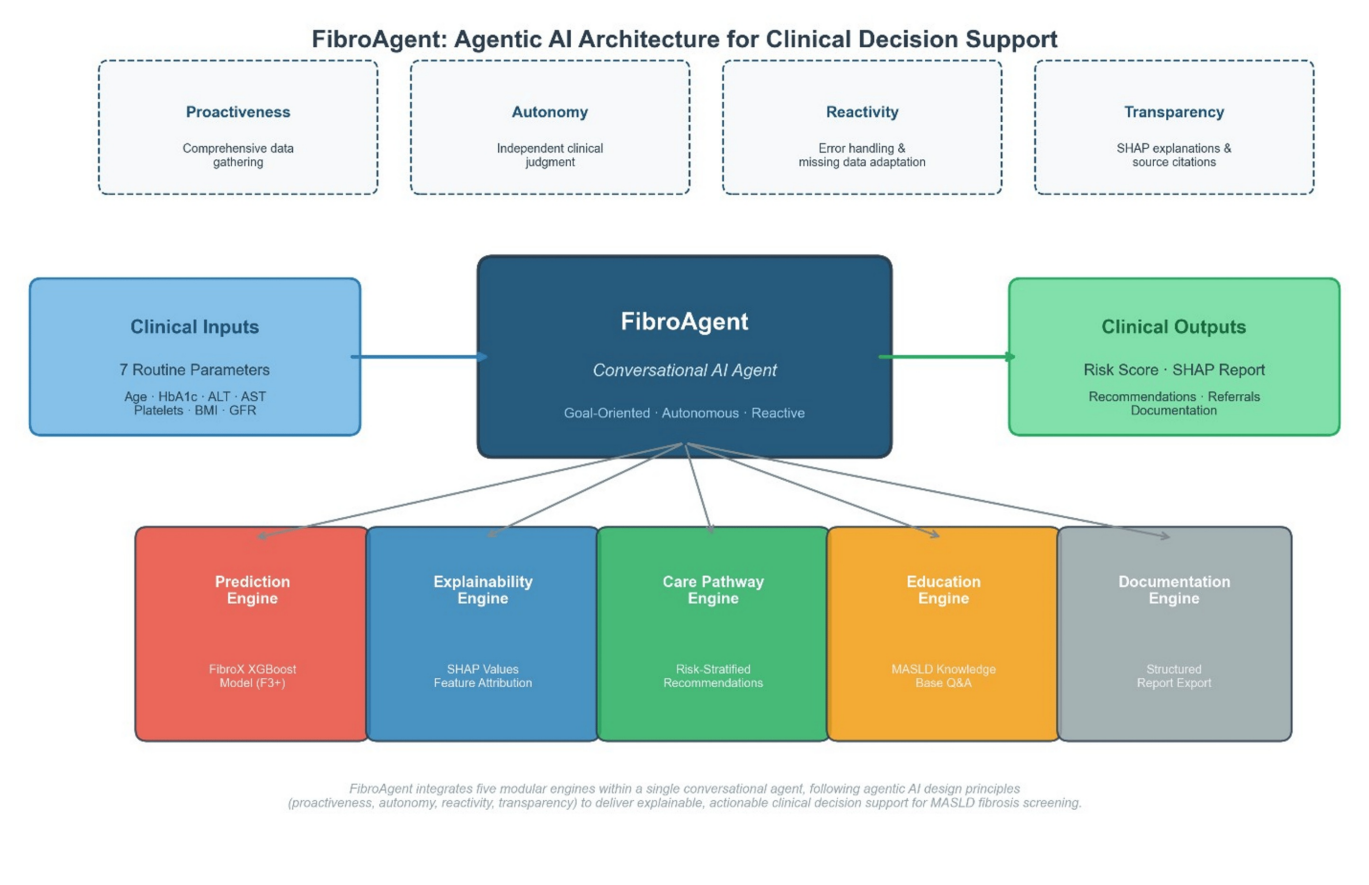
Agentic AI architecture for clinical decision support SHAP: SHapley Additive exPlanations; MASLD: metabolic dysfunction-associated steatotic liver disease

FibroAgent was designed according to the four core principles of an autonomous-agent architecture (Figure 1): proactiveness, autonomy, reactivity, and transparency.

Proactiveness: Rather than passively returning a single risk score, FibroAgent is chartered with the explicit goal of generating comprehensive fibrosis risk assessments that include prediction, explanation, contextualized recommendations, and documentation. The agent proactively gathers and synthesizes all relevant information without requiring the clinician to specify each analytical step.

Autonomy: FibroAgent autonomously orchestrates five modular engines, prediction, explainability, care pathway, education, and documentation, selecting and sequencing the appropriate tools based on clinical context.

Reactivity: The system gracefully handles missing data, invalid inputs, and computational errors. When a component fails, FibroAgent acknowledges the limitation explicitly in its output, continues analysis with available information, and adjusts confidence accordingly.

Transparency: Every prediction is accompanied by SHAP-based feature attributions, clearly indicating which clinical parameters drove the risk assessment and whether each factor increased or decreased risk.

The patient scenarios presented in this study represent illustrative clinical archetypes, and the batch cohort is a simulated dataset constructed to demonstrate system functionality rather than a real-world study population.

System Overview and Capabilities

**Figure 2:**
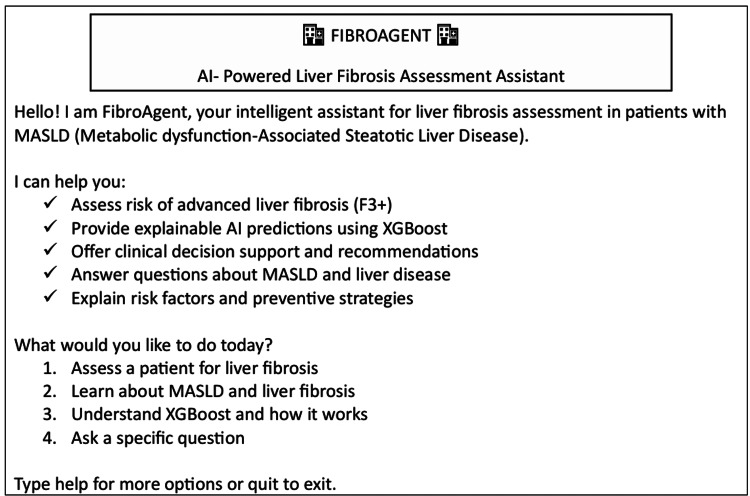
FibroAgent interface and workflow FibroAgent is an AI-powered clinical decision support assistant for assessing advanced liver fibrosis (F3+) in patients with MASLD. The interface summarizes model-based risk stratification, explainable AI outputs, and guideline-aligned clinical recommendations designed to support early fibrosis detection and referral in primary care and general medicine settings. MASLD: metabolic dysfunction-associated steatotic liver disease

FibroAgent is a conversational AI system that integrates multiple functions into a single interface. As demonstrated in the live Jupiter notebook environment (Figure 2), the agent supports structured commands to assess patients, explain predictions, answer disease-related questions, and export reports. The system is built around a validated XGBoost-based model for predicting advanced fibrosis (F3+) in MASLD, augmented with explainability and decision-support layers.

Core capabilities demonstrated include patient risk assessment, SHAP-based explainability, clinical recommendations aligned with risk category, educational knowledge retrieval, batch screening, and the generation of documentation-ready reports.

The prediction engine embedded within FibroAgent is based on a previously developed and validated XGBoost model for advanced fibrosis risk stratification in MASLD; model derivation, cohort characteristics, outcome definition, and performance metrics are reported in the original model publication and were not re-derived in the present proof-of-concept framework study. FibroAgent is currently a proof-of-concept research tool and is not yet publicly released for open access or clinical use without permission.

Inputs, Features, and Minimal Data Requirements

FibroAgent requires seven input parameters: age, glycohemoglobin (HbA1c), alanine aminotransferase, aspartate aminotransferase, platelet count, body mass index, and estimated glomerular filtration rate. These variables were selected to balance predictive performance with real-world feasibility, relying on routinely collected laboratory and anthropometric data commonly available in primary care settings. By avoiding reliance on imaging or specialized biomarkers, FibroAgent is designed to be deployable in low-resource environments where access to elastography or speciality testing is limited.

## Results

Figures were generated programmatically using Python (version 3.14) with Matplotlib and NumPy. Specifically:

Figure 1 (System Architecture): Created using Matplotlib with FancyBboxPatch and annotation functions for the architectural diagram.

**Figure 3:**
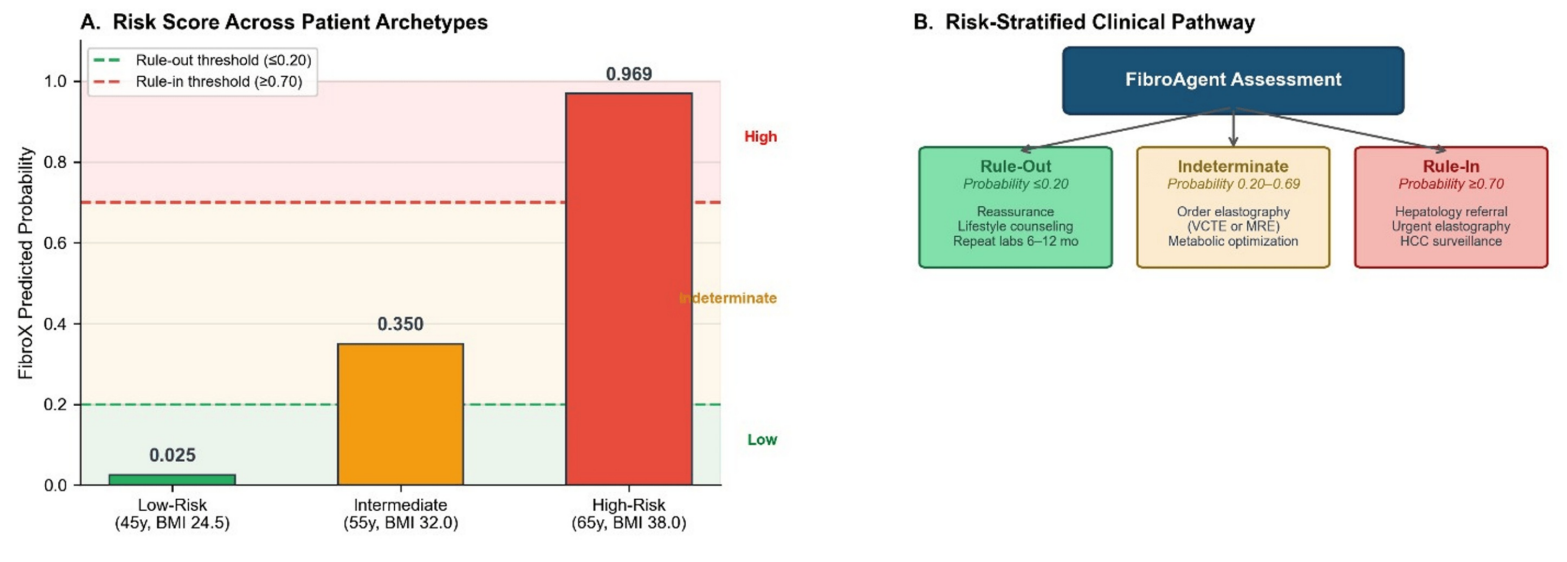
FibroAgent risk stratification

Figure 3 (Risk Stratification): Created using Matplotlib bar charts, gridspec layouts, and FancyBboxPatch elements for the clinical pathway flowchart.

**Figure 4:**
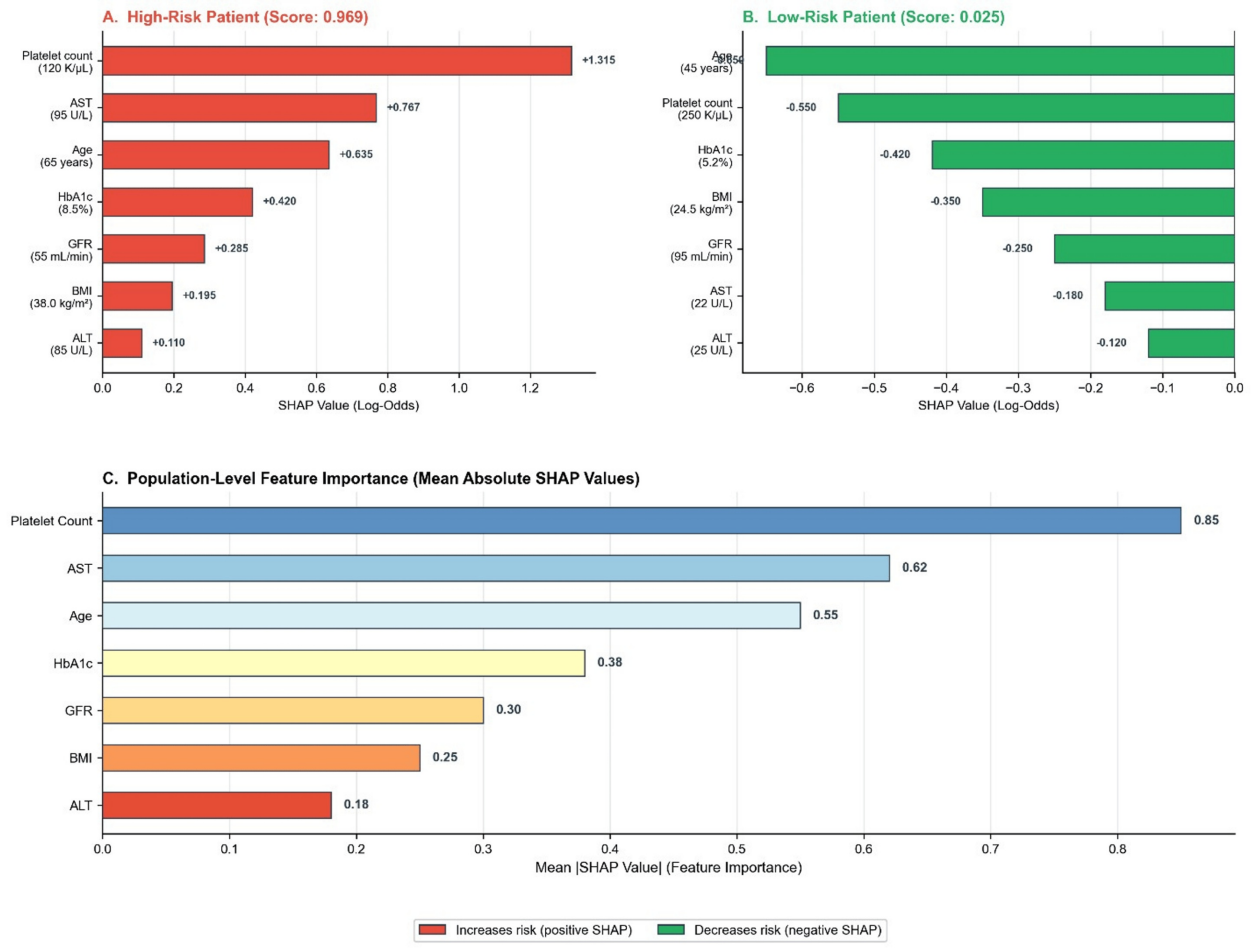
SHAP explainability analysis

Figure 4 (Batch Screening): Created using Matplotlib bar charts and pie charts.

**Figure 5:**
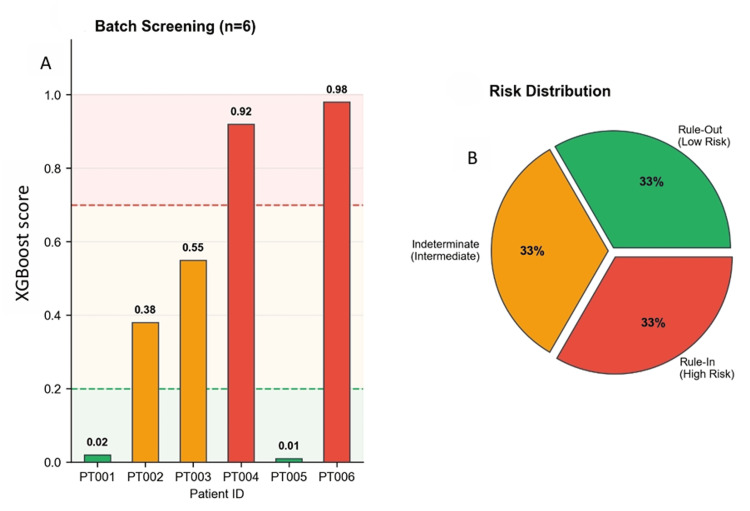
Batch screening demonstration for six patients Panel A: "Traditional Fibrosis Screening (Static Calculator) Panel B: "FibroAgent Agentic CDS (Autonomous Agent)

Figure 5 (Agentic Comparison): Created using Matplotlib with FancyBboxPatch and FancyArrowPatch elements for the workflow comparison diagrams.

Individual patient risk assessment

FibroAgent successfully stratified three clinical archetypes spanning the full risk spectrum (Figure 3): Low-Risk (45-year-old, glycated hemoglobin (HbA1c) 5.2%, alanine aminotransferase 25, aspartate aminotransferase (AST) 22, platelets 250, body mass index (BMI) 24.5, glomerular filtration rate (GFR) 95); Intermediate-Risk (55-year-old, HbA1c 6.5%, ALT 55, AST 48, platelets 180, BMI 32.0, GFR 75), and High-Risk (65-year-old, HbA1c 8.5%, ALT 85, AST 95, platelets 120, BMI 38.0, GFR 55).

Outputs and “human-readable” artefacts

For each assessment, FibroAgent produces a probability-based risk score, assigns a clinically interpretable risk category (rule-out vs rule-in), identifies the most influential predictors using SHAP values, and generates risk-aligned clinical recommendations. These outputs are formatted into a structured report suitable for clinical documentation, including patient parameters, explanation summaries, and actionable next steps. This emphasis on human-readable artefacts distinguishes FibroAgent from traditional calculators that produce outputs without explanation or documentation support.

**Figure 6:**
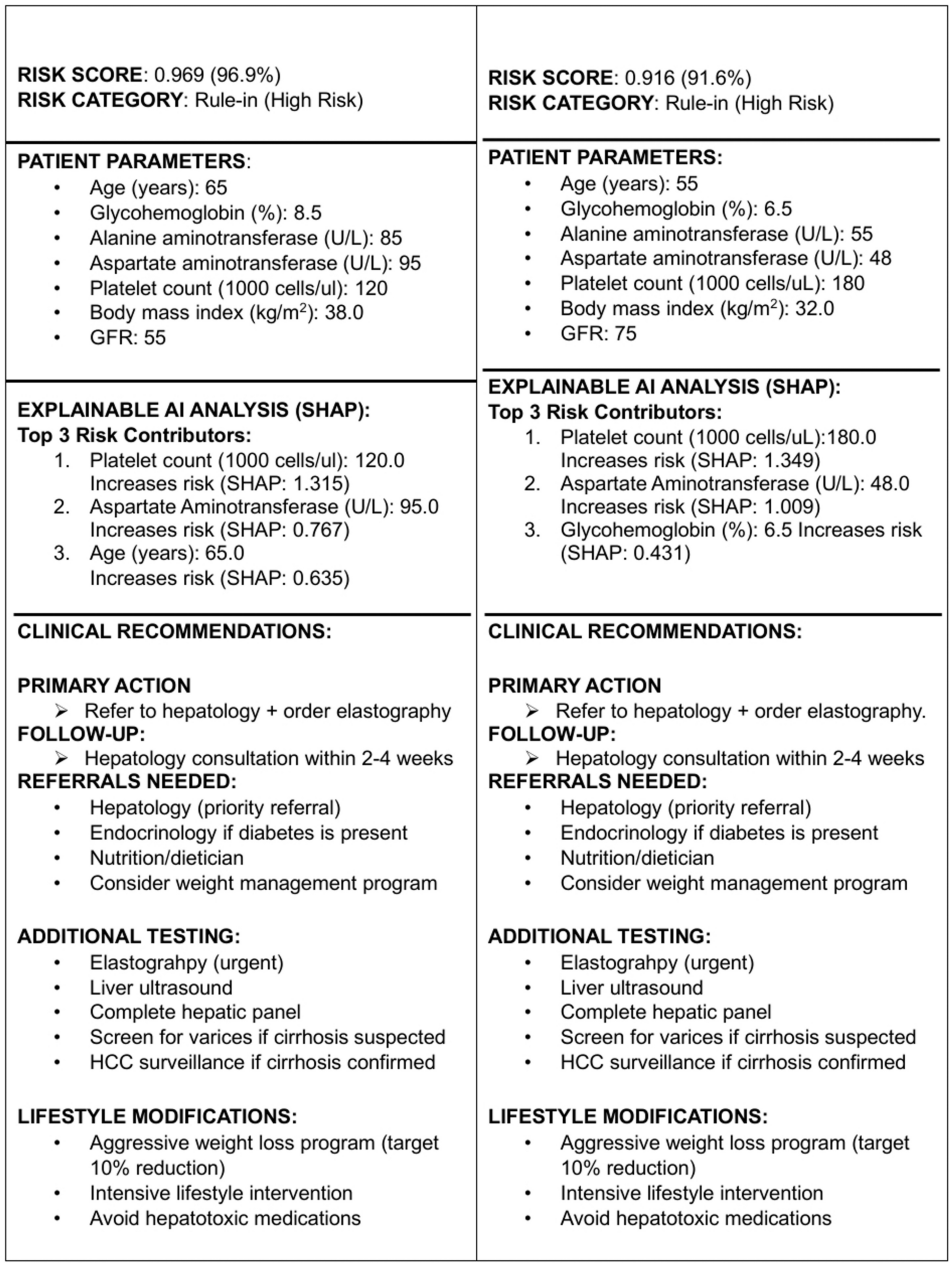
XGBoost risk assessment result 6a- High-risk XGBoost output (rule-in): Example XGBoost assessment demonstrating a high-risk (rule-in) classification for advanced fibrosis (score 0.916). SHAP-based explainability identifies platelet count, AST, and glycohemoglobin as major contributors to risk. The output generates actionable recommendations, including hepatology referral and elastography, illustrating the translation of AI predictions into clinical workflows. 6b- Very high-risk XGBoost output: XGBoost assessment in an older patient with severe metabolic and biochemical abnormalities showing very high risk for advanced fibrosis (score 0.969). Explainable AI highlights platelet count, AST, and age as dominant risk drivers. The tool prioritizes urgent elastography and specialist referral, supporting early identification of patients at high likelihood of advanced disease. SHAP: SHapley Additive exPlanations; AST: aspartate aminotransferase

**Figure 7:**
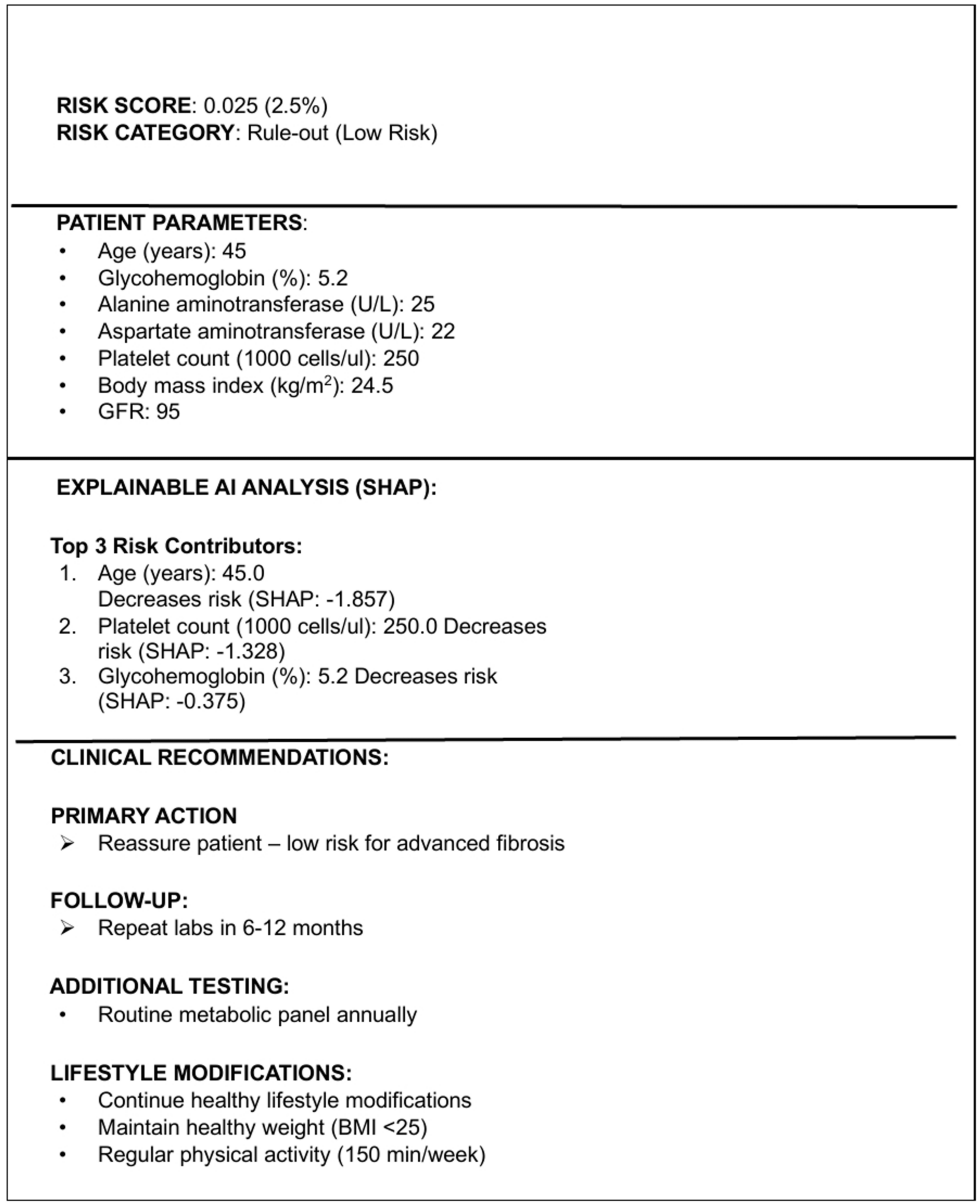
Low-risk XGBoost output (rule-out) Example XGBoost assessment demonstrating a low-risk (rule-out) classification for advanced fibrosis (score 0.025). Protective contributions from a younger age, preserved platelet count, and normal glycohemoglobin are shown via SHAP analysis. The output supports reassurance and routine longitudinal monitoring, reducing unnecessary referrals and testing. SHAP: SHapley Additive exPlanations

Figures 6-7 demonstrate XGBoost assessment outputs. The high-risk patient assessment (Figure 6a) demonstrates a rule-in classification with an XGBoost score of 0.916. SHAP-based explainability identified platelet count, AST, and glycohemoglobin as major contributors to risk, generating actionable recommendations including hepatology referral and elastography. Figure 6b shows an XGBoost assessment in an older patient with severe metabolic and biochemical abnormalities, yielding a very high risk for advanced fibrosis (score 0.969), with platelet count, AST, and age as dominant risk drivers. Figure 7 demonstrates a low-risk (rule-out) classification (score 0.025), with protective contributions from younger age, preserved platelet count, and normal glycohemoglobin identified via SHAP analysis.

SHAP explainability analysis

SHAP-based explanations provided clinically coherent feature attributes across risk categories. In the high-risk patient, all seven features contributed positively to risk, with platelet count, AST, and age demonstrating the largest absolute SHAP values (Figure 4a). In contrast, the low-risk patients showed uniformly negative SHAP contributions, with younger age, preserved platelet count, and normal glycemic control providing the strongest protective effects (Figure 4b).

Population-level feature importance analysis (Figure 4C) revealed that platelet count (mean |SHAP| = 0.85) was consistently the most influential predictor, followed by AST (0.62), age (0.55), HbA1c (0.38), GFR (0.30), BMI (0.25), and ALT (0.18). This hierarchy aligns with established clinical knowledge of fibrosis biomarkers, in which thrombocytopenia reflects portal hypertension and hepatic synthetic dysfunction.

Batch screening demonstration

FibroAgent processed a simulated cohort of 6 patients, automatically classifying patients into risk categories and prioritizing those requiring urgent attention (Figure 5A-6B). Two patients were classified as rule-out (33%), two as indeterminate (33%), and two as rule-in (33%). The system automatically flagged high-risk patients with actionable triage recommendations, demonstrating its feasibility for population-level screening and quality-improvement applications. Figure 5A shows traditional fibrosis screening (Static Calculator), illustrating the conventional workflow in which a clinician enters lab values, FIB-4 calculates a single numeric score, and the clinician must independently interpret, document, and make referral decisions, with no explanation, recommendations, error handling, or documentation support. Figure 5B is a FibroAgent Agentic CDS (Autonomous Agent), illustrating the agentic workflow integrating clinician parameter entry, XGBoost ML prediction with SHAP explanation, risk-stratified clinical pathways, interactive Q&A and patient education, and structured report export with audit trail (Figure 5).

Agentic properties validation

When provided only with patient parameters, FibroAgent proactively generated comprehensive assessments that included risk scores, SHAP explanations, clinical recommendations across five domains (primary action, follow-up, referrals, additional testing, lifestyle modifications), and organised documentation without requiring the clinician to request each component separately. The agent independently coordinated its five engines in the correct order, adjusting the depth and detail of recommendations based on the patient's risk level. High-risk patients received more detailed referral and surveillance suggestions than low-risk patients. When encountering simulated component failures, FibroAgent detected errors, explicitly acknowledged data gaps in the report, continued analysis with available tools, and adjusted confidence levels accordingly, maintaining momentum toward the clinical goal. Every output included complete SHAP-based feature attributions, all input parameters, threshold classification details, and a clinical disclaimer. Generated reports were formatted for audit readiness and care coordination.

## Discussion

FibroAgent represents a meaningful advancement in clinical decision support for liver fibrosis screening by implementing agentic AI principles to address a key gap in hepatology practice. Unlike static risk calculators that generate a single numerical score, FibroAgent offers an integrated, interactive experience that covers the entire clinical workflow, from prediction and explanation to recommendation, education, and documentation. An education and question-answering engine allows users to query topics such as MASLD, fibrosis staging, and explainable AI, while a documentation engine enables export of assessment reports for audit and record-keeping.

Clinical implications

FibroAgent translates fibrosis risk stratification into clear, actionable clinical recommendations, smoothly connecting risk assessment to next steps in care [8]. Patients identified as low risk receive reassurance and guidance for routine follow-up, while those at intermediate or high risk are referred for hepatology consultation, elastography, targeted lab tests, and more intensive lifestyle interventions. This decision logic is transparently encoded and shown within the demo’s recommendation outputs. From a clinician workflow perspective, FibroAgent is used interactively with simple commands such as “assess,” “explain,” and “save,” allowing risk evaluation, interpretation, and documentation to happen in a single session and reducing fragmentation across tools. In this way, FibroAgent is designed to complement existing MASLD care pathways across primary care, endocrinology, obesity medicine, and pre-operative assessments. It functions as an upstream triage and education tool rather than replacing speciality hepatology care [9-11].

Explainability as a clinical tool

SHAP-based explanations serve multiple clinical purposes beyond just transparency [12]. First, identifying patient-specific risk factors enables targeted interventions [7,11]. A patient whose risk mainly results from poor glycemic control (high HbA1c SHAP contribution) might benefit most from diabetes management, while someone with thrombocytopenia may need investigation for portal hypertension [11,13-15]. Second, feature attributions provide a quality assurance mechanism: predictions driven by clinically unlikely factor combinations can be flagged for review, ensuring safe deployment [11,16,17]. Third, explainability encourages patient engagement by translating abstract risk scores into clear, actionable health information [18].

Implementation considerations

As with other clinical decision support (CDS) tools, adopting FibroAgent may face challenges such as alert fatigue, workflow disruption, and clinician trust. To overcome these barriers, FibroAgent emphasizes interaction and explainability, enabling clinicians to actively examine model inputs, intermediate reasoning, and outputs instead of relying on opaque, black-box recommendations. While data availability remains a practical factor, the seven required input variables are commonly collected across most health systems; however, missing data and unit harmonization are foreseeable implementation challenges. Beyond point-of-care use, FibroAgent is intentionally built to support batch processing and population-level screening, allowing the proactive identification of high-risk individuals for population health management and quality improvement efforts. To address documentation and medico-legal issues, the system produces structured, auditable reports that record inputs, predictions, explanations, and recommendations, supporting transparency and defensibility when used as clinician-facing CDS rather than autonomous decision-making.

Governance and safety

FibroAgent is explicitly positioned as a clinical decision-support tool intended to augment, not replace, clinician judgment, with clear disclaimers embedded within both the user interface and generated reports to reinforce appropriate boundaries of use. From a governance perspective, the availability of explainability outputs, including SHAP-based summaries, provides a practical foundation for ongoing auditing, bias detection, and performance surveillance, while periodic recalibration and subgroup-specific performance assessment are proposed as essential requirements for safe real-world deployment [19,20].

Strengths and limitations

The strengths of this study include the demonstration of an integrated agentic clinical decision support framework combining prediction, explainability, clinical recommendations, and documentation within a single system. The study further highlights the feasibility of this approach through representative clinical scenarios and batch screening, with a focus on usability in primary care and resource-limited settings. However, these strengths should be interpreted in the context of several important limitations. FibroAgent is currently demonstrated within a controlled, non-electronic health record (EHR) environment, and real-world integration will inevitably introduce additional technical, workflow, and governance complexity. This controlled setting may not fully capture the variability, interruptions, or operational challenges that occur in routine clinical environments, potentially affecting the tool’s performance and usability. This study represents a proof-of-concept, simulation-based demonstration using clinical archetypes and a simulated cohort, without evaluation in real-world patient populations or prospective or retrospective validation. Because this study was designed as a proof-of-concept demonstration of an agentic CDS framework rather than a model development or validation study, we do not report new discrimination or calibration metrics, and no external validation cohort or gold-standard fibrosis comparator was assessed. In addition, no comparative benchmarking against established fibrosis scores (e.g., FIB-4 or NAFLD fibrosis score) was performed, and the study does not assess clinical impact, including referral accuracy or patient outcomes. Reliance on a fixed seven-variable input set may also limit generalizability across diverse clinical settings and populations, and there remains a risk of clinician over-reliance on automated outputs despite embedded disclaimers and explainability features. This emphasizes that the tool should be interpreted as decision-support rather than definitive diagnostic guidance, and caution is required to avoid overinterpretation of the simulated results.

Future directions

We proposed a phased evaluation strategy for FibroAgent: (1) silent-mode retrospective analysis comparing FibroAgent outputs with usual care; (2) prospective implementation assessing clinical and operational outcomes, including referral patterns, elastography utilization, and diagnostic yield; and (3) user-centered evaluation measuring clinician trust, cognitive load, and workflow integration. Safety monitoring should include false-positive and false-negative rates, as well as subgroup-based equity analyses. Future extensions include EHR integration, automated referral orchestration, retrieval-augmented generation (RAG) for dynamic knowledge updating from practice guidelines, and a multi-agent collaboration architecture where specialized sub-agents handle distinct clinical domains.

## Conclusions

FibroAgent provides a proof-of-concept demonstration of a scalable approach for fibrosis detection in MASLD, combining prediction, explanation, recommendation, education, and documentation in an interactive agent. Based on proven functionality rather than speculative features, FibroAgent shows how explainable, conversational CDS can close the fibrosis detection gap and enable earlier intervention in primary care and resource-limited settings worldwide. These results should be interpreted cautiously, as the study is proof-of-concept and real-world clinical effectiveness has not yet been established. Comparative performance and impact on patient outcomes also require further validation in real-world settings.
